# Alcoholism and Human Electrophysiology

**Published:** 2003

**Authors:** Bernice Porjesz, Henri Begleiter

**Affiliations:** Bernice Porjesz, Ph.D., is an associate professor, and Henri Begleiter, Ph.D., is a professor, both in the Department of Psychiatry and Neuroscience, State University of New York, Downstate Medical Center, Brooklyn, New York

**Keywords:** AOD (alcohol and other drug) dependence, electroencephalography, electrical life processes, evoked potential, P3 amplitude, brain wave, brain imaging, excitatory neurotransmitters, hyperexcitability, disinhibition, neurobiological theory of AODU (alcohol and other drug use), AOD use susceptibility

## Abstract

Electroencephalography (EEG), the recording of electrical signals from the brain, provides a noninvasive measure of brain function as it is happening. Research using EEG, as well as event-related potentials (ERPs) and event-related oscillations (EROs), which measure brain activity in response to a specific stimulus, have shown that the brain activity of alcoholics and nonalcoholics differs in some characteristic ways. These differences are consistent with an imbalance between excitation and inhibition processes in the brains of alcoholics.

Researchers studying the effects of alcohol use on the brain are aided by techniques that yield images of the brain’s structure and reveal the brain’s activity as it is happening. Tools that produce images of the brain’s structure, such as magnetic resonance imaging (MRI) and positron emission tomography (PET), are described in other articles in this issue. This article examines the techniques of electrophysiological brain mapping, which best reveals brain activity as it occurs in time, in fractions of seconds.

Electroencephalography (EEG) is the recording (“graph”) of electrical signals (“electro”) from the brain (“encephalo”). (The resulting report, called an electroencephalogram, also is abbreviated EEG.) Each nerve cell (i.e., neuron) in the brain produces a tiny electrical charge; when a number of neurons become active, the sum of these tiny electrical charges can be detected on the surface of the scalp. Small electrodes placed on the scalp detect this electrical activity, which is magnified and recorded as brain waves (i.e., neural oscillations). These brain waves illustrate the activity as it is taking place in various areas inside the brain.

In the purely resting state, brain waves often are randomly active. However, when a person perceives or responds to a sensory or cognitive stimulus (e.g., a blue triangle), groups of neurons fire together, and the EEG is no longer random. The activity that is related to processing of the stimulus always occurs at the same time after the stimulus (i.e., is time-locked). This time-locked (i.e., event-related or evoked) activity is embedded in background random EEG that is not related to the stimulus processing. In order to see the tiny event-related activity, the EEG is averaged across multiple identical occurrences or trials (e.g., whenever the blue triangle occurs in a series of red squares); activity that is random with respect to the stimulus cancels out with each presentation of the stimulus, whereas the time-locked activity that occurs at the same time on every trial increases in the average. The waveform produced after averaging across identical trials is called an event-related potential (ERP). If one were to make a movie of the brain activity involved in the mental processing of a stimulus as it happens in real time, one would first see early fast activity that is related to sensory reception (e.g., seeing a visual stimulus) occurring in the visual cortex (occipital lobe [see figure 1]); this would then be followed by slower activity related to higher cognitive function (e.g., identification and attention to a blue triangle in a series of squares), which involves activity in the parietal and frontal lobes (figure 1). The fast and slow neural oscillations that underlie the ERP, called event-related oscillations (EROs), represent sensory and cognitive functions.

This article reviews research indicating that alcoholics manifest aberrant resting EEGs, ERPs, and EROs, and discusses the significance of these findings.

## The Resting Electroencephalogram

The resting EEG is the recording of ongoing spontaneous brain electrical activity while the person being examined is relaxing (the person’s eyes may be open or closed). It is made up of oscillations that are described in terms of frequency, which is the number of times a wave completes its cycle per unit of time. Frequency is measured in Hertz (Hz), which is the number of cycles (i.e., undulations) of the wave per second. EEG oscillations also are described by the magnitude of their voltage (power) measured in microvolts (μV [millionths of a volt]).

Typically, EEGs are divided into the following frequency bands: delta (1–3 Hz), theta (3.5–7.5 Hz), alpha (8.0–11.5 Hz), beta (12–28 Hz), and gamma (28.5–50.0 Hz), with each frequency reflecting a different degree of brain activity. In healthy adults, medium (8–13 Hz) and fast (14–30 Hz) frequencies are predominant in the awake resting EEG, with only sparse occurrence of low frequencies (0.3–7.0 Hz) and high frequencies (greater than 30 Hz). The resting EEG is stable throughout healthy adult life and is highly heritable (van Beijsterveldt et al. 1996).

### Theta (3.5–7.5 Hz)

**Figure 1:**
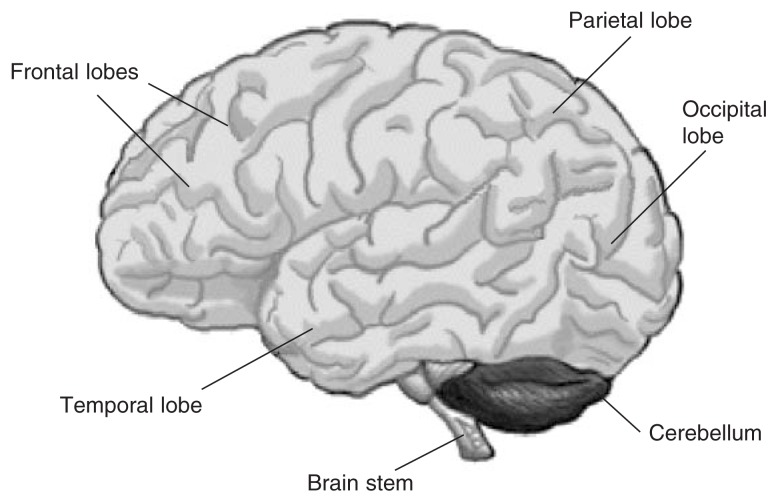
Anatomy of the brain.

Theta rhythm is largest in the back region of the brain when a person is resting (i.e., resting or tonic theta) and in the front region (frontal lobes [figure 1]) when the person is actively engaged in mental activity (i.e., active or phasic theta). The normal adult waking EEG record contains relatively little theta rhythm. Investigators have reported that tonic theta increases in several neurological states, such as Alzheimer’s disease; tonic theta also increases when cognitive activity decreases. Tonic theta power in alcoholics has been examined in the Collaborative Study on the Genetics of Alcoholism (COGA). Researchers compared eyes-closed resting theta (3–7 Hz) power in 307 alcohol-dependent study participants and 307 age- and gender-matched control subjects (Rangaswamy et al. 2003). The alcohol-dependent group had higher resting theta power at all scalp locations. In both male and female alcoholics, the increased theta power was most prominent at parietal regions (parietal regions are located at the top of the brain, in the back; see figure 1); in males this prominent theta also extended more forward to the central regions. Correlation of drinking variables (such as recency of last drink and quantity of drinks in a typical week) with theta power revealed no group-specific differences.

Elevated tonic theta power in the EEG may reflect a deficiency in the information processing capacity of the central nervous system (CNS) (Klimesch et al. 2001). Resting theta power has been reported to increase with age, as well as in Alzheimer’s patients. A theta power increase also may be an electro-physiological indicator of the imbalance in the balance of excitatory and inhibitory neurons in the cortex.

### Alpha (8.0–11.5 Hz)

The alpha rhythm is the predominant EEG rhythm in the relaxed alert person. It is obtained whether eyes are open or closed, and it is strongest over the occipital region (see figure 1) when the person’s eyes are closed. The alpha rhythm is an index of relaxation. Extensive research, dating back to the 1940s, confirms that unstable or poor alpha rhythm is found in alcoholics. Early studies determined that alcoholics manifest less prevalent and lower alpha power than nonalcoholics (for a review, see Begleiter and Platz 1972; Propping et al. 1981), but more recent studies have not been consistent with these findings (Pollock et al. 1992; Enoch et al. 1999).

Enoch and colleagues (1999) have reported that an alpha variant, namely low voltage alpha (LVA), is associated with a subtype of alcoholism that co-occurs with anxiety disorder. Recently, these authors found that among females, LVA is associated with a genetic variant that leads to low activity in catechol-o-methyltransferase (COMT), the enzyme that metabolizes the brain chemicals (i.e., neurotransmitters) dopamine and norepinephrine (NE). The authors hypothesize that the effect of altered NE levels on activity in the thalamus (a communication center in the brain) may partly explain the connections between LVA, anxiety, and alcoholism (Enoch et al. 2003).

### Beta (12–28 Hz)

Beta rhythm is obtained in alert subjects; it is a fast, low-voltage rhythm that is distributed over the scalp. Most studies examining resting EEG characteristics in alcoholics have reported increased beta power in alcoholics compared with nonalcoholics (Bauer 2001; Propping et al. 1981; Winterer et al. 1998). Propping and colleagues (1981) reported these differences in female alcoholics compared with female nonalcoholics, but not in male alcoholics compared with male nonalcoholics. For the COGA study (Rangaswamy et al. 2002), researchers subdivided the beta frequency band into three bands: beta 1 (12–16 Hz), beta 2 (16–20 Hz), and beta 3 (20–28 Hz). The alcohol-dependent group had increased power in both the beta 1 and beta 2 frequency bands at all loci over the scalp, compared with control subjects; this difference was most prominent in the central region of the brain (between the parietal and frontal regions [figure 1]). The alcoholic group also had increased power in the beta 3 frequency band in the frontal region. Age and clinical variables did not influence the difference.

Relapsing alcoholics have been reported to have more desynchronized, or randomly firing, beta activity over frontal areas than nonrelapsers, which suggests a functional disturbance in the prefrontal cortex (the area of the brain just behind the forehead) (Winterer et al. 1998). Bauer (2001) reported that fast beta power was superior to severity of illness, depression level, and childhood conduct problems in predicting relapse in abstinent alcoholics. Researchers have identified anterior frontal brain regions (e.g., the prefrontal cortex) as the most likely source of this fast beta activity. Because the increase in beta power in abstinent alcoholics was not related to length of abstinence (Rangaswamy et al. 2002) and also is present in offspring of alcoholics at risk for alcohol dependence (Rangaswamy et al. 2004), these findings suggest that excess beta power is a “trait” rather than a “state” variable (i.e., related to underlying genetic predisposition and not to alcohol use or other factors). This is consistent with the hypothesis that an imbalance between excitatory and inhibitory neurons is involved in a predisposition to develop alcohol dependence (Begleiter and Porjesz 1999) as well as a proneness to relapse (Bauer 2001).

Beta rhythm reflects a balance between networks of nerve cells projecting from the cortex to other parts of the brain and spinal cord (i.e., pyramidal cells) (which are excitatory) and neurons that carry signals between other neurons (i.e., interneurons) (which are inhibitory). GABA_A,_ the receptor, or binding molecule, for the neurotransmitter gamma-aminobutyric acid (GABA), is thought to regulate this rhythm (Whittington et al. 2000).

A recent finding from the COGA project has found a genetic linkage (within families) and linkage disequilibrium (across families) between the beta frequency of the EEG and a GABA_A_ receptor gene (i.e., they co-occur at a higher frequency than would be predicted by chance) (Porjesz et al. 2002). Furthermore, several neuroimaging studies of alcoholics have shown deficits in the GABA receptors for the chemical benzodiazepine, which facilitates inhibitory GABAergic transmission (Abi-Dargham et al. 1998; Lingford-Hughes et al. 1998). Researchers also have reported neuronal loss or shrinkage in the superior frontal and motor cortices of alcoholics (Harper and Kril 1993). Taken together, these findings suggest that this deficit in GABA receptors in the brains of alcoholics may account for their lack of CNS inhibition (i.e., hyperexcitability). Recent findings from the COGA project indicate that the same GABA_A_ receptor gene associated with the beta frequency of the EEG also is associated with a diagnosis of alcohol dependence, based on criteria from the *Diagnostic and Statistical Manual of Mental Disorders, Fourth Edition* (DSM–IV) (Edenberg et al. 2004). This suggests that variations in the GABA_A_ receptor 2–subunit gene (*GABRA2*) (the gene encoding the alpha 2 subunit of the GABA_A_ receptor) affect the level of neural excitability, which in turn affects the predisposition to develop alcohol dependence.

### Event-Related Potentials (ERPs)

**Figure 2:**
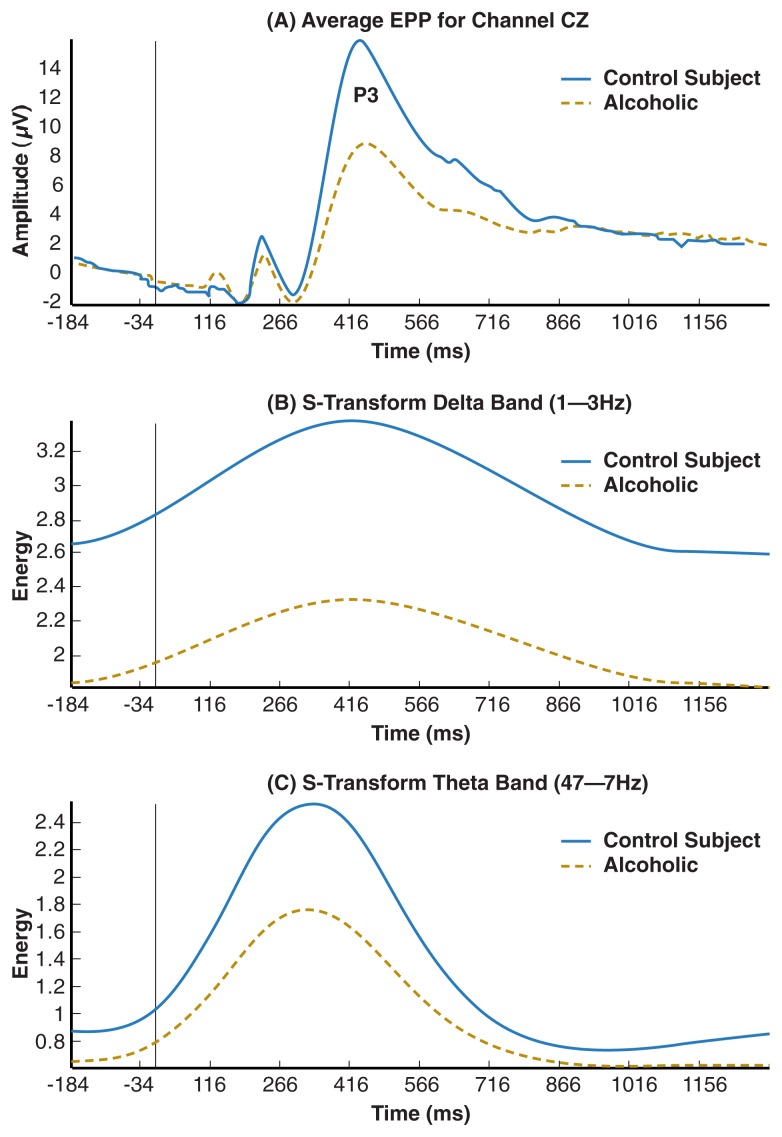
**(A)** Average ERP waveform in response to a target stimulus in a visual oddball task. Recorded at the midline central electrode in a group of alcoholics (red) and a group of control subjects (blue). Note that the P3 component is reduced in alcoholics compared with control subjects. **(B)** Time–energy curve in the delta frequency band during the processing of a target stimulus for control subjects (blue) and alcoholics (red). Note the reduced delta power in alcoholics compared with control subjects. **(C)** Time–energy curve in the theta frequency band during processing of a target stimulus for control subject (blue) and alcoholics (red). Note the reduced theta power in alcoholics compared with control subjects.

Unlike the resting EEG, which is a recording of ongoing brain activity, ERPs reflect brain electrical activity in response to specific sensory or cognitive events occurring at a specific time. ERPs can be used to monitor brain activity ranging from sensory reception to higher cognitive processes. ERPs are characteristic, highly reproducible waveforms that are displayed graphically (figure 2A) as a series of peaks (designated P for positive components) and valleys (designated N for negative components). Components are described in terms of their wave height (amplitude, measured in microvolts [μV]), and the time of their occurrence following presentation of the stimulus (latency, measured in milliseconds [ms]). For example, P300 is a positive peak occurring around 300 ms after the stimulus; it also is designated as P3 (i.e., third positive peak). Early components, those with a latency of less than 100 ms, reflect responses to the physical characteristics of the stimulus, whereas later components are influenced by more cognitive factors.

### P300 (P3a, P3b)

Most studies investigating electrophysiological deficits in alcoholics have focused on the P300 or P3 component. The P3 is a large positive component that occurs between 300 and 700 ms after a “significant” stimulus and is not related to the physical features of the stimulus (e.g., brightness and shape for visual stimuli, or loudness and pitch for auditory stimuli). A stimulus can be “significant” by being relevant to a task (e.g., the subject must press a button whenever a specific stimulus, such as a blue triangle, occurs), by having a motivating influence (e.g., the subject wins money after responding to the stimulus correctly), or by occurring rarely or unpredictably. The P3 is thought to reflect aspects of working memory, the temporary storage of information required for complex cognitive tasks such as learning, reasoning, and comprehension. Specifically, P3 may reflect attention allocation and updating processes (Polich and Herbst 2000). P3 also is thought to reflect cognitive closure, or the termination of a mental process (Desmedt 1980; Verleger 1988), which involves inhibition over widespread cortical areas (Desmedt 1980; Verleger 1988; Rockstroh et al. 1992; Birbaumer et al. 1990; Tomberg and Desmedt 1998). The amplitude of P3 reflects inhibition of responses to irrelevant stimuli that the subject must ignore in order to respond effectively to the relevant targets (Desmedt 1980; Birbaumer et al. 1990; Klimesch et al. 2000). The timing of P3 occurrence (latency) reflects mental processing speed (Polich and Herbst 2000); the earlier and larger the P3, the easier the processing.

The ERP task most commonly used to elicit the P3 is the so-called “odd-ball” task, in which rare “oddball” stimuli are embedded in a series of frequent stimuli (standards or nontargets). For example, in an auditory task, the subject listens to frequent “boops” and rare “beeps” in a random stream of tone bursts. If the subject is asked to attend or respond to the rare “beep” stimulus, it is designated as a target; the P3s recorded in response to these task-relevant targets are largest posteriorly on the scalp (over the parietal region [figure 1]) and are designated as P3b components. If the subject is not asked to attend to rare “beep” stimuli, P3s recorded to these unattended rare nontarget stimuli in a repetitive background have a more frontal distribution and are designated as P3a (figure 1); although rare nontargets elicit these P3as, frequent nontargets usually do not elicit any P3s.

Studies over the last few decades have found that the amplitudes of P3s to task-relevant target stimuli (P3b) are significantly lower in abstinent alcoholics than in nonalcoholics (for reviews, see Porjesz and Begleiter 1996, 1998). This deficit in alcoholics occurs with both auditory and visual tasks but is seen more consistently with visual tasks. More recent studies have indicated that low P3 amplitudes are present not only in male alcoholics but also in female alcoholics, though not to the same extent as in males. Figure 2A illustrates reduced P3 amplitudes in alcoholics, compared with control subjects, in response to target stimuli in a visual paradigm.

Not only do alcoholics manifest low amplitude P3b components in response to target stimuli, they also manifest low P3a components in response to rare nontargets in both visual and auditory modalities. Recent reports have indicated that alcoholics manifest reduced P3 amplitudes to both Go (target) and No-Go (rare nontarget) stimuli. Furthermore, alcoholics manifest less differentiation between their responses to target and nontarget stimuli. In keeping with various neurophysiological explanations of the P3 component (Desmedt 1980; Verleger 1988; Birbaumer et al. 1990; Rockstroh et al. 1992; Klimesch et al. 2000), the amplitude of P3 is thought to reflect CNS inhibition (the larger the P3, the greater the inhibition). An increase in theta power, an inhibitory rhythm, underlies P3 (see the following section on event-related oscillations). Most information a person is exposed to is irrelevant and must be suppressed while the person selectively responds to the relevant information; this accounts for the large amplitude of the P3 (Klimesch et al. 2000). The low-amplitude P3 components manifested by alcoholics indicate that they have less CNS inhibition than control subjects. Researchers have hypothesized that this lack of inhibition, or underlying CNS disinhibition (i.e., hyperexcitablity), is involved in a predisposition to alcoholism (Begleiter and Porjesz 1999).

As expected, nonalcoholics manifest their largest P3b amplitudes in response to targets over parietal regions of the scalp (figure 1), and their largest P3a amplitudes in response to rare nontargets. However, alcoholics manifest similar low-amplitude P3s across all areas of the scalp in response to rare target and nontarget stimuli.

Despite P3’s maximal amplitude over parietal areas in response to targets when scalp electrodes are used, studies with electrodes inserted into the brain (i.e., depth electrodes) in humans indicate that P3s originate in the frontal cortex; the hippocampus, which is important in the consolidation of new memories; and the amygdala, a part of the limbic system involved in producing and controlling emotional behavior. Recent functional magnetic resonance (fMRI) studies support these findings and implicate another part of the limbic system, the anterior cingulate area of the frontal cortex, as critical for P3 generation. The lower amplitude P3 components, along with the weaker and less well organized sources in alcoholics, suggest disorganized and inefficient brain functioning. This global neurophysiological pattern suggests cortical disinhibition, providing further support for underlying CNS hyperexcitability in alcoholics.

## Event-Related Oscillations (EROs)

The neural oscillations that underlie ERPs are called EROs. Although EROs are measured in the same frequency bands as spontaneous resting EEGs— namely, delta (1–3 Hz), theta (3.5–7.5 Hz), alpha (8.0–11.5 Hz), beta (12–28 Hz), and gamma (28.5–50.0 Hz)—functionally they are different from spontaneous resting EEG rhythms. EROs temporally are related to the sensory and cognitive processing of stimuli (Basar et al. 1999). During sensory reception, groups of neurons that are close together fire together at fast rates in the gamma range. Cognitive processing (e.g., attention to an auditory rather than a visual stimulus), however, involves communication between brain regions that are somewhat farther apart (e.g., adjacent temporal and parietal lobes [figure 1]). This processing involves synchronization between the brain regions in the alpha and beta frequency ranges. Higher cognitive processing (e.g., working memory, determining if a stimulus has been seen before) involves interactions between widely separated brain regions (e.g., frontal and parietal lobes [figure 1]). Higher cognitive processing involves slow synchronization in the theta or delta frequency range (Lubar 1997). Thus, faster frequencies represent synchronization of groups of neurons in more local areas, whereas slower frequencies are involved in synchronization over longer distances in the brain (von Stein and Sarnthein 2000; Kopell et al. 2000).

### Theta/Delta Underlying Visual P3

As discussed above, P3 has multiple sources, with contributions from the frontal cortex and hippocampus. P3 consists of delta and theta oscillations with a higher proportion of delta oscillations from the posterior regions of the brain, and theta occurring in the frontal and central regions (Yordanova and Kolev 1996; Basar et al. 1999; Basar-Eroglu et al. 1992; Karakas et al. 2000). Synchronization occurs in the theta range between the hippocampus and frontal and parietal regions in the brain (figure 1) (i.e., these regions communicate with each other) during attention tasks.

Slow EEG oscillations (delta and theta) depend on the activity level of specific receptors (i.e., muscarinic cholinergic receptors) for the brain chemical acetylcholine; the level of acetylcholine in the cortex and hippocampus is reduced during delta oscillations and elevated during theta activity. Recent findings from the COGA project indicate that the cholinergic receptor genes are involved in theta and delta production (Jones et al. in press) and that these genes also are associated with alcohol dependence. The production of theta and delta rhythms also involves interactions between the GABA and cholinergic neurotransmitter systems; the frequency of theta is controlled by the GABA system, whereas its power is controlled by the cholinergic system (Fellous and Sejnowski 2000; Tiesinga et al. 2001).

In a visual oddball paradigm, evoked delta and theta power while processing the target stimuli is significantly decreased among alcoholics compared with control subjects (see figures 2B and 2C), indicating that the reduced P3 amplitudes reported in alcoholics are caused by deficits in theta and delta oscillations that underlie P3. In a Go/No-Go paradigm, alcoholics also manifested significantly decreased delta and theta power, particularly during No-Go processing (Kamarajan et al. 2004). An increase in theta power is related to an increase in theta in the hippocampus, known to be an inhibitory rhythm associated with GABAergic activity (Klimesch et al. 2000). An increase in theta is associated with inhibition of nonrelevant information while attending to relevant information (e.g., a target stimulus). As most information is irrelevant and must be suppressed, this yields the high amplitude of P3 to relevant stimuli. Hence the deficit in inhibitory theta oscillations underlying P3 in alcoholics suggests deficient inhibitory control during information processing (e.g., attention and memory mechanisms) in alcoholics. This finding provides further support for the hypothesis that CNS disinhibition is involved in alcoholism (Begleiter and Porjesz 1999).

### Frontal Midline Theta

Frontal midline theta observed in humans during attention-related tasks is integral to understanding brain functioning during information processing. In one study, researchers examined event-related EEG changes during the performance of mental calculations in alcoholics and control subjects. The study was based on the hypothesis that performance deficits would be indicated by reduced frontal theta power in alcoholic patients. Researchers recorded EEGs in adult alcoholics and normal volunteers during the performance of a simple addition problem (active theta) and during a resting interval (resting theta). The difference between resting and active theta power is a measure of processing capacity—the lower the resting theta power and the higher the active theta power, the more efficient the brain processing (Klimesch et al. 2001). The difference in theta power between resting and problem-solving conditions was significantly lower in alcoholics compared with control subjects at the anterior frontal electrodes. Alcoholics manifested increased resting theta and decreased active theta, indicating decreased and inefficient processing capacity. These deficits in performance, indexed by low evoked (active) theta power during mental effort, reflect frontal lobe dysfunction in alcoholics (Suresh et al. submitted [*a*]). These deficits involve inhibitory processes and are manifested as impairments in working memory and sustained attention.

### Gamma

Numerous studies indicate that gamma oscillations serve as a mechanism for binding features of an object (feature binding)—for example, the shape and color of an object. Early phase-locked gamma is involved in selective attention and is larger in response to attended stimuli than unattended stimuli, particularly over frontal regions (Basar et al. 1999; Yordanova et al. 2001). Several investigators have reported an association between gamma oscillations and P3 components obtained in response to target stimuli in an oddball task (Basar et al. 1999). One recent study found that alcoholics manifest lower gamma power (29–45 Hz) than control subjects during target processing between 0 and 150 ms in a visual oddball paradigm. This effect was strongest frontally and lateralized to the left side. No group differences in gamma were observed for nontarget and novel stimuli. Control subjects manifested significantly higher gamma power in the processing of the target relative to the processing of the nontarget stimulus, whereas alcoholics did not manifest higher gamma power during target processing. Increased evoked gamma is thought to reflect a matching process between the template in working memory and the current stimulus. These findings of gamma deficits in response to target stimuli in alcoholics, particularly in frontal regions, provide further evidence for deficits in cognitive processes (e.g., attention allocation, working memory) in alcoholics.

## Summary and Conclusions

One of the most consistent electrophysiological measures that characterize male alcoholics is the low amplitude of their P3 components; female alcoholics also manifest reduced visual and auditory P3 amplitudes, although to a lesser extent than male alcoholics. Recent findings indicate that not only are P3 components at low amplitude in alcoholics, but the neural oscillations underlying P3 are deficient in alcoholics as well. The findings that both evoked delta and evoked theta oscillations underlying P3 are deficient in alcoholics imply dysfunction in both the cognitive processes and neural correlates that mediate these oscillations. Event-related delta oscillations are related to signal detection (i.e., stimulus discrimination of one object from all other objects) and decisionmaking, whereas theta oscillations are associated with cognitive processes such as attention, alertness, and memory (Basar et al. 1999). Alcoholics are also deficient in the production of evoked gamma oscillations during the processing of target stimuli. As these oscillations are related to selective attention processes and working memory, these findings indicate that alcoholics manifest deficits in cognitive functions associated with these oscillatory processes.

Alcoholics are deficient not only in their response to task-relevant target stimuli (P3bs) but also in response to task-irrelevant rare stimuli (P3a), as revealed by ERP findings. Recent ERO findings indicate that alcoholics manifest decreased theta and delta oscillations to both Go and No-Go stimuli in a Go/No-Go task. As increased theta power indicates inhibition of responding to irrelevant information during selective information processing tasks (Klimesch et al. 2000), decreased theta oscillations in alcoholics may reflect deficient inhibitory control.

During oddball tasks, control subjects manifest enhanced P3 components and evoked theta, delta, and gamma oscillations when processing the target stimulus but not when processing a nontarget or novel stimulus. Alcoholics, however, manifest less electrophysiological differentiation among the three stimulus categories. Topographic maps of P3s and these EROs during target processing indicate that not only do alcoholics manifest weaker sources, they have less topographically distinct spatial–temporal patterns. This less differentiated mode of responding during various tasks indicates that alcoholics are less proficient at processes which involve comparing a new stimulus to a template, suggesting alcoholics have attention and memory deficits. Alcoholics seem less able to efficiently use available information (e.g., a template in working memory) to respond differentially to incoming stimuli (targets, nontargets); hence each incoming stimulus must be evaluated anew. This more global mode of responding in alcoholics regardless of stimulus and task requirements indicates a basic diminution of differential inhibition. In healthy people, familiar stimuli are processed with less neuronal activity than unfamiliar stimuli. Evidence from monkey studies indicates that repeated stimuli elicit less neuronal firing than novel stimuli, suggesting inhibition of masses of neurons (Miller et al. 1991), which leads to increased synaptic efficiency. Differential inhibition allows the animal to efficiently process a given stimulus (e.g., target). Thus, reduced differential neuronal inhibition of relevant and irrelevant stimuli in alcoholics may account for the electrophysiological aberrations observed in alcoholics.

For many years it was assumed that the P3 deficit observed in alcoholics was the consequence of the deleterious effects of alcohol on the brain. However, after a sufficient period of abstinence, many of the clinical abnormalities characteristic of alcohol dependence, as well as electrophysiological measures of hearing deficits, return to normal, but the P3 amplitude abnormality persists (Porjesz and Begleiter 1985). This protracted deficit in long-term abstinent alcoholics suggests the possibility that P3 deficits may precede alcohol use and dependence. Indeed, a number of studies have reported low P3 amplitudes in young people at high risk for developing alcoholism, such as young sons of alcoholic fathers (Begleiter et al. 1984; Polich et al. 1994).

Recent findings indicate that in addition to P3, many of the aberrations in resting and event-related oscillations reported in alcoholics already are apparent in high-risk offspring of alcoholics before alcohol exposure. The increased resting beta power observed in alcoholics also is present in both male and female offspring of alcoholics (Rangaswamy et al. 2004). The frontal theta deficits observed in alcoholics during a mental calculation task also are apparent in offspring of alcoholics (Suresh et al. submitted [*b*]). Similar to their alcoholic parents, the offspring manifest increased resting theta and decreased active frontal theta, suggesting that cognitive processing may be inefficient in these people before the development of alcoholism. As the electrophysiological differences are not related to length of abstinence and are apparent in people at risk before they have been exposed to alcohol, these neural oscillations could be considered markers of risk. The electrophysiological imbalances in excitation–inhibition observed in the offspring of alcoholics may be involved in the predisposition to develop alcoholism (Begleiter and Porjesz 1999). Long-term studies of childhood and adolescent precursors of adult alcohol abuse consistently identify a cluster of behavioral traits described as disinhibited, undercontrolled, impulsive, or aggressive, which significantly predict high levels of alcohol consumption or abuse.

Taken together, the electrophysiological findings suggest that an imbalance between excitation and inhibition (i.e., CNS disinhibition or hyperexcitability) may be involved in a predisposition to develop alcoholism. Alcoholics and people at risk for alcoholism manifest increased resting oscillations (e.g., theta, beta) and decreased “active” oscillations in the same frequency bands during cognitive tasks. Not only does this underlying CNS disinhibition appear to be involved in the predisposition toward alcoholism (Begleiter and Porjesz 1999), but it also is hypothesized that neuroelectric features related to CNS disinhibition may provide insights into the neurobiology of craving and relapse. The relationship between this underlying CNS hyperexcitability and the induction of alcohol abuse leading to alcohol dependence remains to be explained.
